# Effect of Hydrogen Addition on Coke Formation and Product Distribution in Catalytic Coupling of Methane

**DOI:** 10.1021/acs.iecr.4c00381

**Published:** 2024-04-09

**Authors:** Rolf S. Postma, Leon Lefferts

**Affiliations:** Catalytic Processes and Materials Group, Faculty of Science and Technology, MESA+ Institute for Nanotechnology, University of Twente, PO Box 217, Enschede 7500 AE, The Netherlands

## Abstract

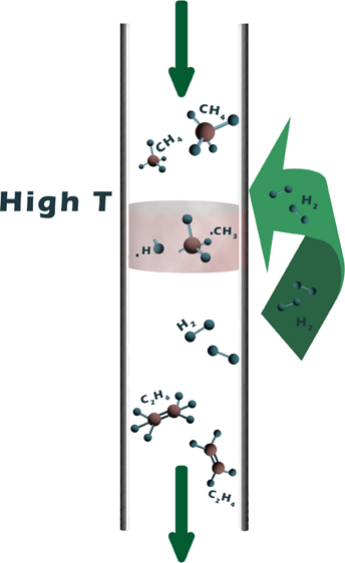

The effect of hydrogen addition on catalytic nonoxidative coupling of methane at 1000 °C was investigated. Experiments were performed at varying ratios between the catalyst and the postcatalytic volume to discern the effect of hydrogen on the catalytic reaction as well as on the gas-phase reaction. Adding 10% H_2_ decreases the methane conversion by a factor of 2, almost independent of the ratio between the catalyst and the postcatalytic residence time. The effect on the conversion is mostly determined by gas-phase chemistry. Hydrogen addition has no influence on the C_2_ hydrocarbon yield, whereas aromatic selectivity is significantly reduced. Changes in selectivity are attributed to changes in methane conversion. Quantitative determination of the amount of coke deposited on the catalyst reveals a decrease by 1 order of magnitude when dosing up to 10% H_2_, while carbon deposits-downstream of the catalyst bed are suppressed to a much lower extent. These results suggest a process in which the produced hydrogen is partly recycled, maximizing the carbon selectivity to C_2_ hydrocarbons while minimizing both aromatics and, most crucially, formation of coke on the catalyst as well as further deposits-downstream.

## Introduction

Methane is receiving growing interest as chemical feedstock for the production of liquid fuels and base chemicals,^1−3^ such as olefins and aromatics. The traditional role of methane-rich natural gas for heating and electricity production is expected to be taken over by renewable sources such as solar or wind,^4−7^ making natural gas and methane from anaerobic digestion of biomass available for synthesis of chemicals. Most processes for methane valorisation to higher hydrocarbons rely on steam reforming of methane to synthesis gas, followed by various processes to produce paraffins, olefins, or aromatics.^8,9^

Direct catalytic nonoxidative coupling of methane has seen a steep increase in interest over the past decade.^3,10,11^ The more traditional routes for direct methane coupling, namely, oxidative coupling of methane (OCM)^12^ and methane dehydroaromatization,^13^ suffer from low single pass conversions and low selectivity caused by formation of CO_2_ and coke, respectively. In 2014, Guo et al.^14^ reported coupling of methane over a Fe©SiO_2_ catalyst to a mix of olefins and aromatics at temperatures above 950 °C. They reported single pass hydrocarbon yields as high as 48% at 1080 °C, without coke formation and long-term stability up to 60h. The absence of coke formation has since only been reproduced by Sakbodin et al.^15^ who tested the same catalyst in a hydrogen permeable membrane reactor. Other publications report coke formation during methane coupling using the Fe/SiO_2_ catalyst.^16−20^ Han et al.^17^ reported that SiO_2_ in the cristobalite phase, as well as atomically dispersed iron sites are crucial for minimizing coke formation. Our earlier work^19^ as well as a Sabic patent^20^ showed that coking can be minimized by using a small amount of catalyst to initiate the reaction by forming free radicals, while most of the CH_4_ conversion occurs in the postcatalytic volume via gas-phase radical reactions. It should be noted though that others suggested surface reactions contribute to coupling reactions as well.^21,22^ Furthermore, our earlier work^19^ showed that rapid heating upstream of the catalyst bed, preventing methane activation in the gas phase, also minimizes coking.

Research on noncatalytic direct coupling of methane, generally called methane pyrolysis, surged in the 80s and 90s.^23−28^ Most of these studies focused on unravelling the free-radical gas-phase mechanism in direct methane coupling. Hydrogen was occasionally used as the coreactant, slowing down the free radical reactions and preventing excessive deposit formation.^27,29−32^ Guéret et al.^31^ showed that hydrogen addition at ratios of H_2_:CH_4_ between 0.5 and 2 decreases methane conversion and dehydrogenation rates of both ethylene and acetylene, as well as decreases the rate of cyclization to benzene at 1330 °C. They attribute all these effects to scavenging of CH_3_· radicals. Olsvik et al.^32^ investigated the effect of H_2_ addition at temperatures between 1200 and 1500 °C, showing that hydrogen addition prevents excessive carbon formation. Arutyunov reviewed a large set of data on methane pyrolysis, spanning from 1862 up to 1990,^33^ including the work of Germain et al.,^34^ who demonstrated that dilution of methane with hydrogen significantly decreases the methane conversion rate, whereas dilution with nitrogen had no significant effect. In contrast, Kunugi et al.^35^ showed that addition of relatively small concentration of hydrogen, less than 20%, increases the methane conversion rate at temperatures above 1300 °C. Kim et al.^36^ trained a neural network with a large set of experimental data on methane pyrolysis to determine the optimum reaction conditions, namely, pressure, temperature, flow-rate, H_2_ cofeeding, reactor length, and reactor diameter. Hydrogen concentration was found to be the main determining factor in methane conversion as well as C_2_ selectivity, and the optimum hydrogen concentration was calculated at 3.6 vol % at a reaction temperature of 1200 °C.

There are sparse data on the effect of hydrogen addition on the catalytic coupling of methane. Sakbodin et al.^15^ showed the effect of H_2_ addition up to 16.7% during catalytic methane pyrolysis using Fe/SiO_2_ as the catalyst. Methane conversion halved on the addition of 9.1% hydrogen to the reactant stream, while the selectivity to C_2_ products increased significantly and selectivity to aromatics decreased. Interestingly, the C_2_ yield remains relatively unchanged, whereas the aromatic yield drops significantly. Note that Sakbodin et al. did not report any coke formation. Similar experiments using Pt/Al_2_O_3_ catalysts for dehydrogenative coupling of methane^37^ show the same trend of decreasing methane conversion and increasing C_2_ selectivity upon 10% H_2_ addition. Coke selectivity was significantly suppressed, leading to long turn (300h+) catalyst stability as opposed to rapid deactivation when no hydrogen was cofed.

This study reports on the effect of hydrogen addition on catalytic nonoxidative coupling of methane, including experimental determination of the amount of coke on the Fe/SiO_2_ catalyst as well as making distinction between deposits on the catalyst versus formation of deposit-downstream of the catalyst bed. Also, the residence time at high temperature downstream of the catalyst bed is varied in order to distinguish between the influence of hydrogen on catalytic reactions and gas-phase reactions.

## Experimental Section

### Catalyst Synthesis

The catalyst is synthesized according to the method described in ref (14), while details concerning catalyst synthesis as well as characterization can be found in ref (19). In short, in-house synthesized Fe_2_SiO_4_ is mixed with quartz and ball-milled in a N_2_ atmosphere overnight using a zirconia milling jar. The resulting powder is fused for 6 h at 1700 °C in air. The resulting slab is crushed and sieved; the fraction between 250 and 500 μm is used in the experiments. The sieved catalyst particles are leached for 2h in 0.5 M HNO_3_, rinsed, and dried to obtain the final catalyst. The catalyst is analyzed with both XRD and XRF, showing the expected cristobalite crystal structure and 0.5 wt % Fe loading. For more details concerning the catalyst characterization, please refer to our previous work.^19^

### Reactor Setup

**Figure 1:**
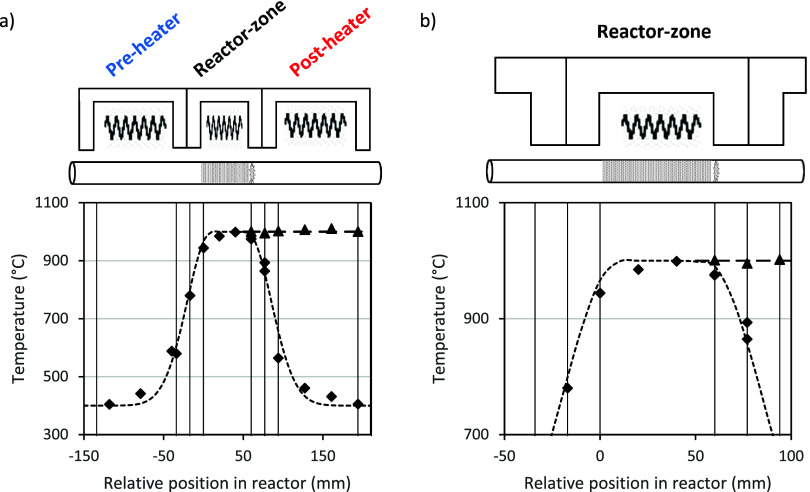
(a) Temperature profile inside the reactor measured with an empty reactor tube. (b) Zoom in on (a); gas-flow rates of 10 mL/min N_2_; vertical bars represent the insulating layers between the three different zones. ◆ Pre- and postheater at 400 °C; reactor zone at 1000 °C; ▲ Postheater at 1000 °C. Identical to ref (19) and repeated here for clarity.

A modular three-zone oven is used for catalytic testing, as reported in our earlier work.^19^ Each zone is thermally insulated from the others and from the environment, allowing for steep temperature gradients, as presented in Figure 1. The preheater is always operated at 400 °C and the reactor zone at 1000 °C; the postheater is either operated at 400 or 1000 °C, as shown by the two separate lines in Figure 1. Gas flow rates are controlled with mass flow controllers. Product gases are analyzed using a three-channel Varian CP-3800 in-line gas chromatograph, and the tubing between the reactor and the GC is heated to 200 °C to prevent hydrocarbon condensation. Further details are presented in ref (19).

### Experimental Procedure

**Figure 2:**
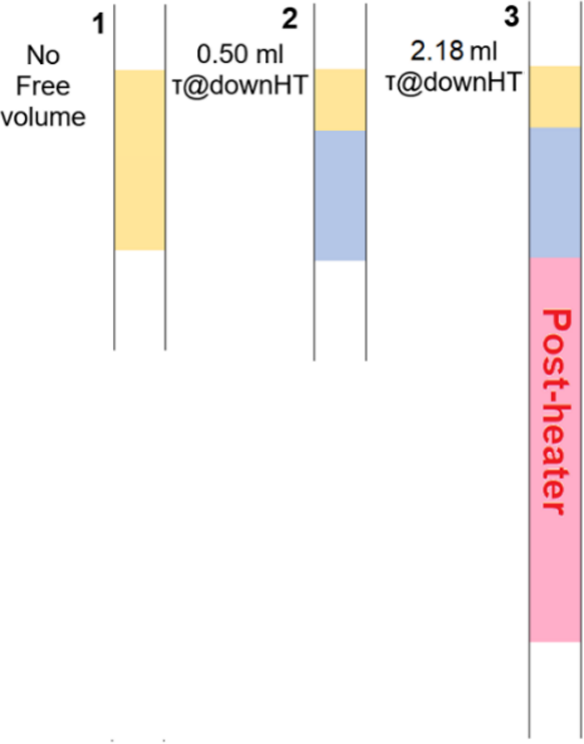
Three positioning of the catalyst inside the 6 cm zone of oven segment 2. Case 1 operated with a catalyst bed height of 6 cm (820 mg), leaving no free volume at reaction temperature; case 2 contains a 2 cm high bed (260 mg) at the top of the 6 cm reactor zone; case 3 is similar to situation 2 but with the postheater at the same temperature as the reactor zone.

The experimental procedure is similar to previous work.^19^ The catalyst is placed at the desired position in the quartz reactor with an inner diameter of 4 mm, according to Figure 2, and held in position by a small quartz wool plug. Three different catalyst positions are considered; in case 1, the complete reactor zone (Figure 1) is filled with the catalyst, minimizing free volume at high temperature (*T* > 950 °C). In both cases 2 and 3, only the top 2 cm of the 6 cm reactor zone is filled with the catalyst. The reactor zone is heated at 1000 °C without using the postheating in case 2, resulting in 0.5 mL free volume at a high reaction temperature. In contrast, in case 3 (Figure 2) the postheater (Figure 1) is also heated to 1000 °C, resulting in 2.2 mL of free volume at reaction temperature downstream of the catalyst. Radial temperature gradients are assumed insignificant. The reactor is flushed for 10 min with 10 mL/min N_2_. The catalyst is heated with 20 °C/min to 900 °C under 10 mL/min of N_2_. The catalyst is then exposed to 90 vol % CH_4_ in N_2_ at 1000 mL·g_cat_^–1^·h^–1^ during 2 h for activation, following the procedure of Bao c.s.^38^ After activation, the reactor is flushed for 10 min at 900 °C with 10 mL of N_2_ at the rate of 10 mL/min. The temperature is increased to 1000 °C, with a heating rate of 20 °C/min, and the experiment is started by feeding 90 vol % CH_4_ balanced with N_2_. Hydrogen is added while reducing the amount of nitrogen in the feed, keeping the methane concentration as well as the total flow rate constant. The total space velocity was kept constant at 2440 mL·h^–1^·g_cat_^–1^ unless otherwise noted. At the end of the experiment, the reactor is flushed for 10 min with N_2_ at 10 mL/min, after which the reactor ovens are turned off and left to cool, maintaining the N_2_ flow while cooling. The catalyst is removed at the end of the experiment and analyzed for coke deposition using TGA (Mettler Toledo TGA/DSC 3+ Star System), heating the sample at 10 °C/min to 1000 °C in 1:1 air/Ar; for more details, refer to ref (19). The online GC sampled every 27 min, and all data presented are based on at least three measurements in steady-state conditions.

Methane conversion is calculated according to
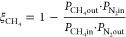
1

: conversion of methane (−); *P*_X_: partial pressure of compound X (bar).

Methane conversion is corrected for any change in the molar flow rate based on the change in the nitrogen tracer concentration according toeq 1.^14,39^ Selectivity is calculated on molar carbon base, corrected for any change in the molar flow rates based on the concentration of the N_2_ tracer, eq 2,^14,39^ adjusting for stoichiometry.
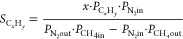
2

: selectivity toward C_*x*_H_*y*_ hydrocarbon (−), *x* refering to the carbon number.

The total carbon-to-product selectivity is the summation of selectivity to all products, as shown in eq 3.
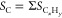
3

*S*_c_: total carbon to products selectivity (−).

Integral calculations of conversion, selectivity, and coke deposition are made in order to compare with the data on the amount of deposits on the catalyst as measured with TGA. Eq 4 calculates the total amount of carbon converted in the full experiment:
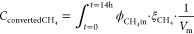
4

: flow of *x* (mL·s^–1^); *V*_m_: molar volume at SPT (mL·mol^–1^).

The total amount of carbon that is converted to deposits during an integral experiment is calculated based on the difference in mass-balance closure, as presented in eq 5:
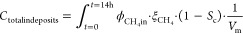
5

Deposits form either on the catalyst, named “coke-on-catalyst”, or downstream of the catalyst bed on the reactor wall and in the tubing to the GC, named “deposits-downstream”. The amount of coke formed on the catalyst during a full experiment is calculated based on the weight loss during oxidation of the spent catalyst in TGA, according to eq 6, assuming that the deposits contain exclusively *C*:
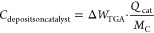
6

*M*_C_: molar mass of carbon (g·mol^–1^); Δ*W*_TGA_: the percentage of mass lost in the TGA analysis (−); *Q*_cat_: amount of catalyst loaded in the reactor (g).

The remaining gap in the mass balance is attributed to the formation of “deposit-downstream” in the system downstream of the catalyst bed. Deposits include a wide array of polyaromatic hydrocarbons of C_10+_ in carbon number, as well as coke deposited on the reactor wall downstream of the catalyst, as presented in a previous work.^19^

## Results

**Figure 3:**
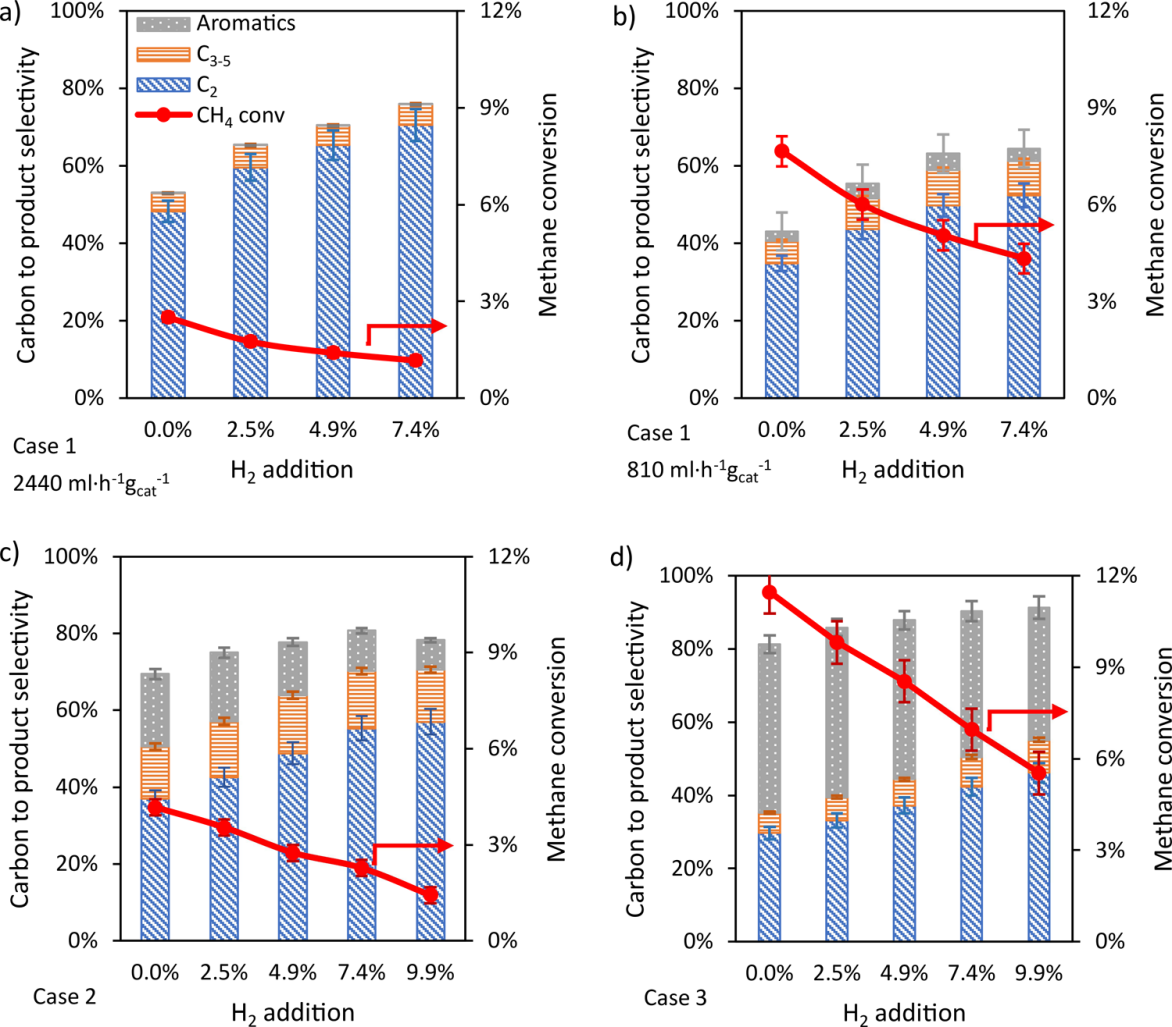
Effect of hydrogen addition on methane conversion and carbon-to-product selectivity, 90% CH_4_, N_2_ balance; reactor zone at 1000 °C and preheater at 400 °C; (a) case 1, large catalyst bed, postheater at 400 °C, space velocity: 2440 mL·h^–1^·g_cat_^–1^, flow rate of 33.3 mL·min^–1^; (b) case 1, large catalyst bed, postheater at 400 °C, space velocity: 810 mL·h^–1^·g_cat_^–1^, flow rate of 11.1 mL·min^–1^; (c) case 2, small catalyst bed, postheater at 400 °C, space velocity: 2440 mL·h^–1^·g_cat_^–1^, flow rate of 11.1 mL·min^–1^; (d) case 3, small catalyst bed, postheater at 1000 °C, space velocity: 2440 mL·h^–1^·g_cat_^–1^, flow rate of 11.1 mL·min^–1^.

Figure 3 shows the effect of hydrogen concentration in the reactant mixture for the three different cases of operating the reactor, as shown in Figure 2. Note that Figure 3a,b shows the results for case 1 at different space velocities, while Figure 3a presents the results obtained with the same space velocity as used for (c) and (d); i.e., the flow rate for (b) and (c) was decreased with a factor of 3. The result reported in Figure 3b was obtained using the same low flow rate as (c) and (d). SI Figure S1 shows more detailed results on the product distribution.

In general, the total carbon-to-products selectivity increases significantly when dosing small amounts of hydrogen, while at the same time, methane conversion significantly decreases. Especially, the selectivity of C_2_ hydrocarbons increases on H_2_ addition. The increase in carbon-to-product selectivity is especially strong when using a large catalyst bed (case 1, Figure 3a,b). A large catalyst bed causes a low selectivity to aromatics, which increases significantly on increasing the postcatalytic volume (cases 2 and 3, Figure 3c,d) in accordance with our earlier work.^19^ The addition of hydrogen causes a significant decrease in selectivity to aromatics in that case (Figure 3c,d).

**Figure 4:**
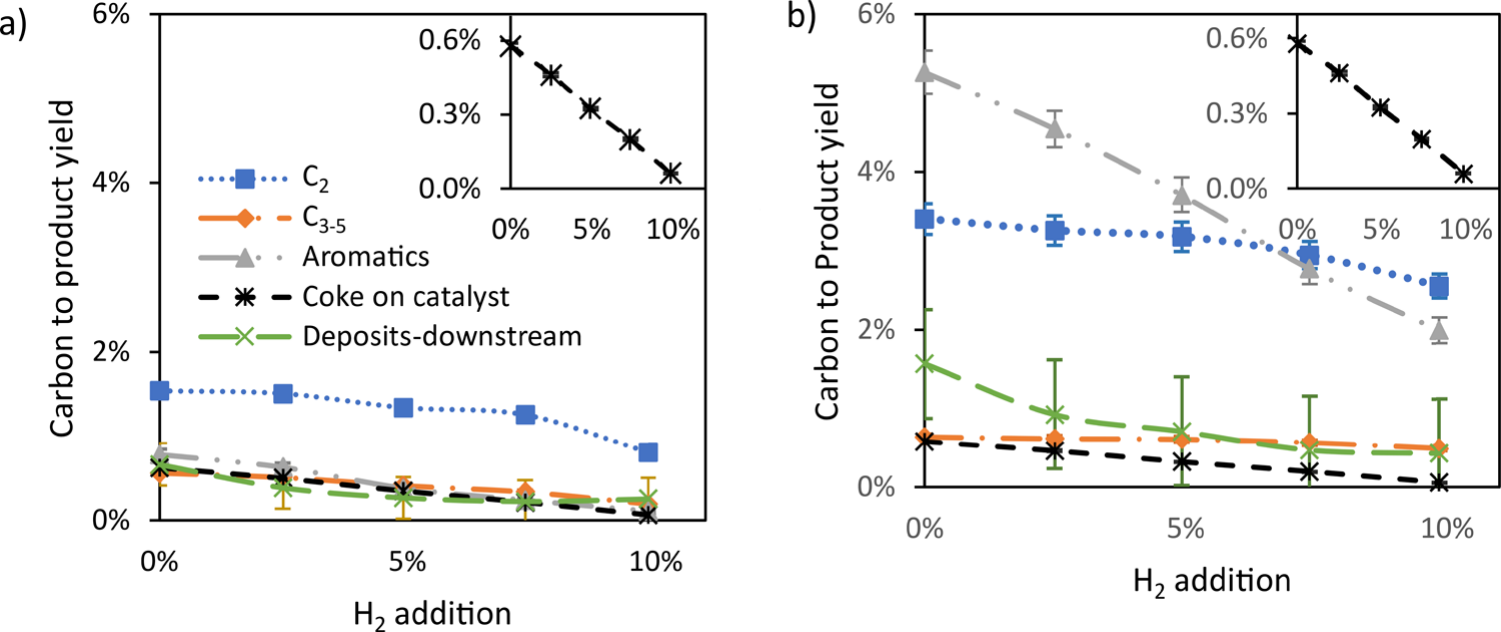
Effect of hydrogen addition on C-yield distribution over hydrocarbon products, coke-on-catalyst (TGA basis), and deposits-downstream of the catalyst (based on the mass balance); reactor zone at 1000 °C, preheater at 400 °C, space velocity: 2440 mL·h^–1^·g_cat_^–1^; (a) case 2 with a small catalyst bed and postheater at 400 °C; (b) case 3 with a small catalyst bed with postheater at 1000 °C. Note that the inset on “coke-on-catalyst” in Figure 4b is assumed to be identical to Figure 4a, as described in the text.

Figure 4 shows the product yield distributions, including the formation of coke on the catalyst and deposits, for experiments with a small catalyst bed. Figure 4a presents the results obtained with a small catalyst bed without a postheater (case 2 in Figure 2), corresponding to the results in Figure 3c. Figure 4b presents results operating with the post-heater (case 3 in Figure 2), corresponding to the data in Figure 3d. Measurements during 6 h were conducted for every hydrogen concentration presented in Figure 4a without a postheater, in order to determine the quantity of coke-on-catalyst formed via TGA, presented in SI Figure S3b. It is reasonable to assume that the quantity of coke is also valid for operation with postheater (Figure 4b) since the conditions in the postheater cannot influence coke formation on the catalyst. This assumption is supported by the observation that the amount of coke on the catalyst formed, as determined with TGA (SI Figure S3a), is very similar during the experiments, resulting in the data presented in Figure 3c,d; the amount of coke-on-catalyst without and with postheater is 8.58 and 8.35 mg, respectively. The formation of deposits-downstream in Figure 4 is estimated based on the mass balance as described in the experimental section. Hydrogen addition causes a decrease in the yield of all products. The decrease is more significant for products with a higher carbon number, with the increase being most significant for coke formation on the catalyst. In contrast, the decrease in C_2_ hydrocarbon yield is relatively small.

Reductive coke removal was attempted in a separate experiment, showing that coke removal in pure H_2_ at reaction conditions (i.e., 1000 °C) proved impossible due to thermodynamic constrains.^2^

## Discussion

**Figure 5:**
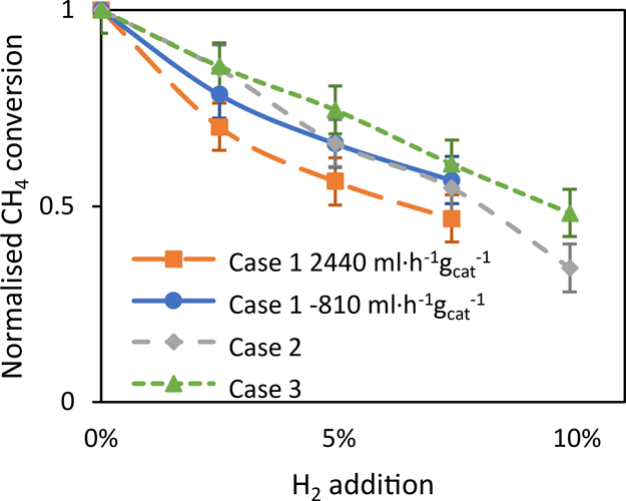
Normalized reduction in methane conversion as a function of H_2_ addition presented in Figure 3. Case 1 uses a reactor zone fully filled with the catalyst at 1000 °C having the pre- and postheater at 400 °C at two different space velocities, 810 and 2440 mL·h^–1^·g_cat_^–1^. Case 2 uses only the top third of the reactor zone filled with the catalyst at 1000 °C, the pre- and postheater at 400 °C, at a space velocity of 2440 mL·h^–1^·g_cat_^–1^ and case 3 uses only the top third of the reactor zone filled with catalyst, reactor zone and postheater at 1000 °C and the preheater at 400 °C, at a space velocity of 2440 mL·h^–1^·g_cat_^–1^.

Methane conversion is significantly reduced when hydrogen is added to the reactant mixture. Figure 5 shows that the relative decrease in methane conversion is slightly larger in the case of a large catalyst bed, i.e., case 1 compared to operating with a small catalyst bed (cases 2 and 3). On the other hand, the differences are not very large, suggesting that the effect of hydrogen is mainly caused by reactions in the gas phase. Similar observations were made by Germain and Vaniscotte^33,34^ as well as Guéret and Billaud^31^ in the absence of a catalyst, attributing the decrease in methane conversion to scavenging of the formed CH_3_· radicals by hydrogen. The observed 50% decrease of methane conversion by adding typically 7.5–10% hydrogen (Figure 5) is in line with the observations by Sakbodin et al.^15^

Figure 4 shows a minor decrease of the yield of C_2_ hydrocarbons, while conversion decreases significantly with adding H_2_, implying that the product distribution shifts strongly toward C_2_ hydrocarbons, in agreement with the observations by Sakbodin et al.^15^ The selectivity toward C_3–5_ hydrocarbons remains stable on hydrogen addition, while selectivity to aromatics decreases as can be seen in Figure 3. For clarity reasons, the same data are plotted in Figure S2 in the SI, clearly showing the trends in selectivity.

**Figure 6:**
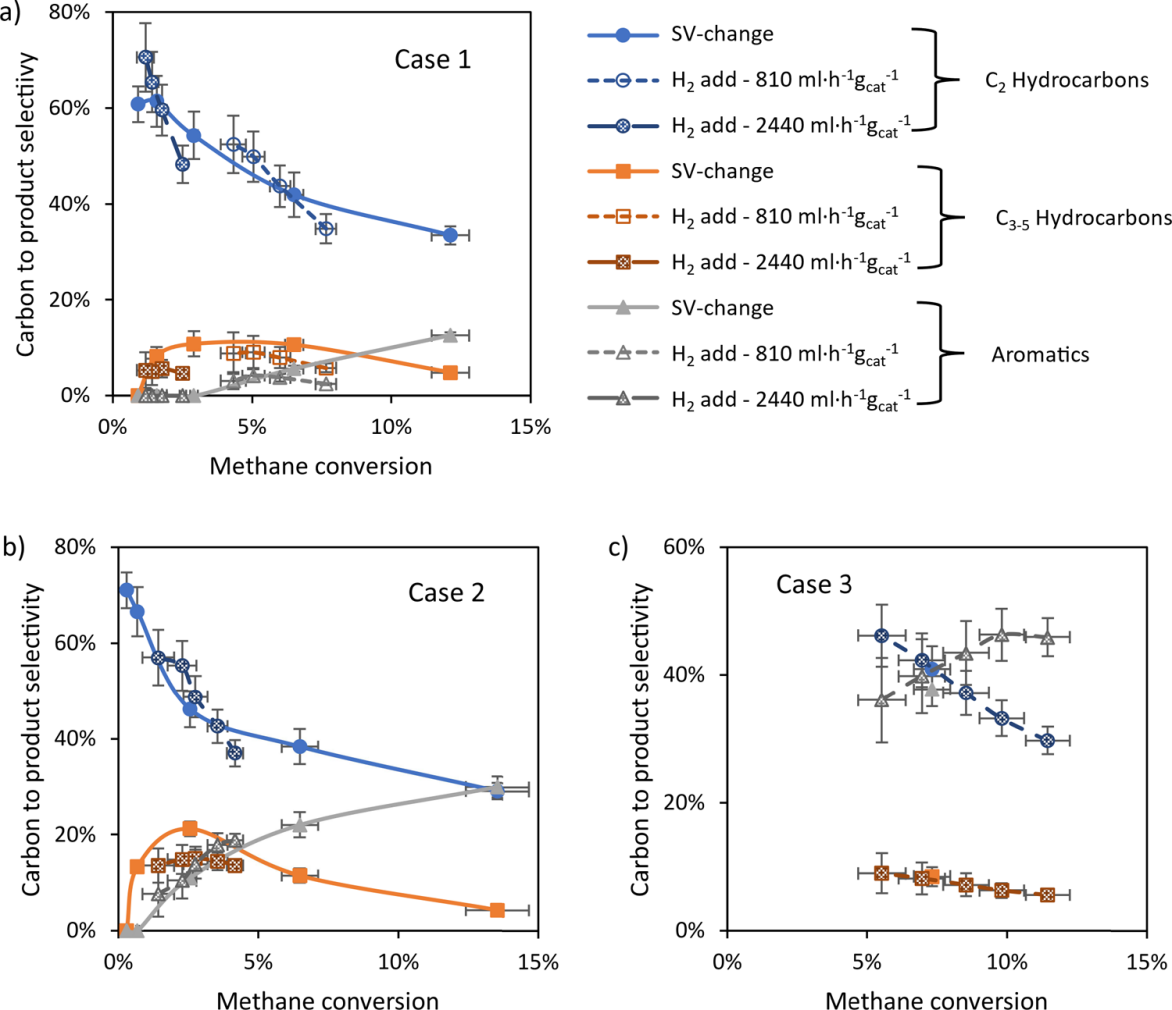
Selectivity distribution over major product groups as a function of methane conversion and varying methane conversion via either hydrogen addition or space velocity increase. All data on the effect space velocity change without hydrogen addition have been adopted from an earlier publication.^19^ Reactor zone at 1000 °C, preheater at 400 °C; (a) case 1 with a large catalyst bed at both space velocities (2440 and 810 mL·h^–1^·g_cat_^–1^), postheater at 400 °C with hydrogen addition between 0 and 7.4 vol %; space velocity variation between 4060 and 240 mL·h^–1^·g_cat_^–1^ without hydrogen addition; (b) case 2, small catalyst bed, postheater at 400 °C, space velocity 2440 mL·h^–1^·g_cat_^–1^ with hydrogen addition between 0 and 9.9 vol %; space velocity between 13,200 and 800 mL·h^–1^·g_cat_^–1^ without hydrogen addition; (c) case 3, small catalyst bed, postheater at 1000 °C, space velocity of 2440 mL·h^–1^·g_cat_^–1^ with hydrogen addition between 0 and 9.9 vol %; without hydrogen addition a single measurement at space velocity of 4000 mL·h^–1^·g_cat_^–1^.

Figure 6 compares the selectivity to carbon-to-product as a function of methane conversion on adding different concentrations of hydrogen, as well as on varying the space velocity without hydrogen addition. In this way, it is possible to discuss the effect of hydrogen addition on selectivity at constant methane conversion, similar to our previous work.^19^ The change in selectivity when adding hydrogen is, within experimental error, identical to the change in selectivity when changing the conversion via the space velocity. This signifies that the change in product distribution on hydrogen addition is mainly a consequence of the lower methane conversion level. This is different from the results of Guéret and Billaud,^31^ reporting that the scavenging of the formed CH_3_· radicals reduces all reaction rates involving these radicals, resulting in stabilization of C_2_H_4_ and slowing down the coupling reactions of C_2_H_2_ to C_4_ and C_6_ hydrocarbons. The difference is probably due to the higher temperature and H_2_ partial pressure used in their work, 1300 °C and 33–66 vol % H_2_, respectively.

Hydrogen addition significantly reduces the formation of coke on the catalyst, up to an order of magnitude, at 10% hydrogen addition, as shown in Figure 4. Tomono et al.^37^ also proposed similarly decreasing coke formation without actually measuring the amount of carbon deposits. Coke formation on the catalyst requires deep dehydrogenation of precursors, e.g. acetylene and aromatics,^40^ which is suppressed by increasing the hydrogen pressure. Deposit formation downstream of the catalyst, estimated based on the mass balance (Figure 4), also decreases but to a lesser extent. We suggest that this is because hydrogen is a product of the reaction and is therefore always present in the gas mixture downstream of the catalyst bed. Adding hydrogen therefore increases the hydrogen concentration relatively much stronger at the beginning of the catalyst bed than further downstream. Therefore, suppression of coke formation on the catalyst is stronger than suppression of formation of deposits-downstream. The coke formed on the catalyst cannot be removed with hydrogen at the reaction temperatures, as experimentally observed. Thus, the decrease in the amount of coke-on-catalyst is caused by suppression of formation.

Hydrogen addition can be a potent tool to control coke formation while maintaining C_2_ yield in nonoxidative coupling of methane by reducing coking tendencies at the inlet to the catalyst bed. Hydrogen is a product of the NOCM reaction and can thus be recycled back into the reactor, which would also simplify hydrogen purification. Hydrogen recycling can thus be highly valuable for designing a nonoxidative route for direct ethylene synthesis from methane, maximizing carbon utilization.

## Conclusions

The effect of hydrogen addition on coke formation and product distribution in direct catalytic coupling of methane to higher hydrocarbons was quantitatively investigated, varying the residence time at a high temperature downstream of the catalyst bed. It was found that hydrogen addition up to 10% in the reactor feed, when operating the reactor at 1000 °C, decreases methane conversion by a factor of 2. This decrease in methane conversion is dominated by reactions in the gas phase, which is known to be caused by methyl radical scavenging by the added hydrogen. The selectivity to hydrocarbon products increases significantly, which is largely attributed to the decrease in the methane conversion level. Remarkably, formation of coke deposits on the catalyst decreased 1 order of magnitude. Formation of deposits-downstream of the catalyst is suppressed by a factor of 2 only. The yield of light olefins remained unaffected by the addition of hydrogen. Recycling part of the produced hydrogen may offer a process scheme with high olefin yields while minimizing formation of coke on the catalyst, deposits-downstream of the catalyst, and higher aromatic products.

## Supporting Information Available

Additional information on product distributions and TGA analysis of spent catalysts (PDF)
