# Supplementary material for: Effect of Hydrogen Addition on Coke Formation and Product Distribution in Catalytic Coupling of Methane
Source: Ind Eng Chem Res. 2024 Apr 9;63(16):6995–7002. doi: 10.1021/acs.iecr.4c00381 (PMC11046431; doi:10.1021/acs.iecr.4c00381)
Supplement: Supplementary file 1 — ie4c00381_si_001.pdf [file ie4c00381_si_001.pdf]

Supporting information

# The effect of hydrogen addition on coke formation and product distribution in catalytic coupling of methane

Rolf S. Postma[a,b], Leon Lefferts[a,*]

[a] Catalytic Processes and Materials Group, Faculty of Science and Technology, MESA+ Institute for Nanotechnology, University of Twente, PO Box 217, Enschede, 7500 AE, Netherlands

[b] Present address: Stamicarbon B.V., Mercator 3, 6135 KW, Sittard, Netherlands

[*] L.Lefferts@utwente.nl

## S.1 Detailed product distribution

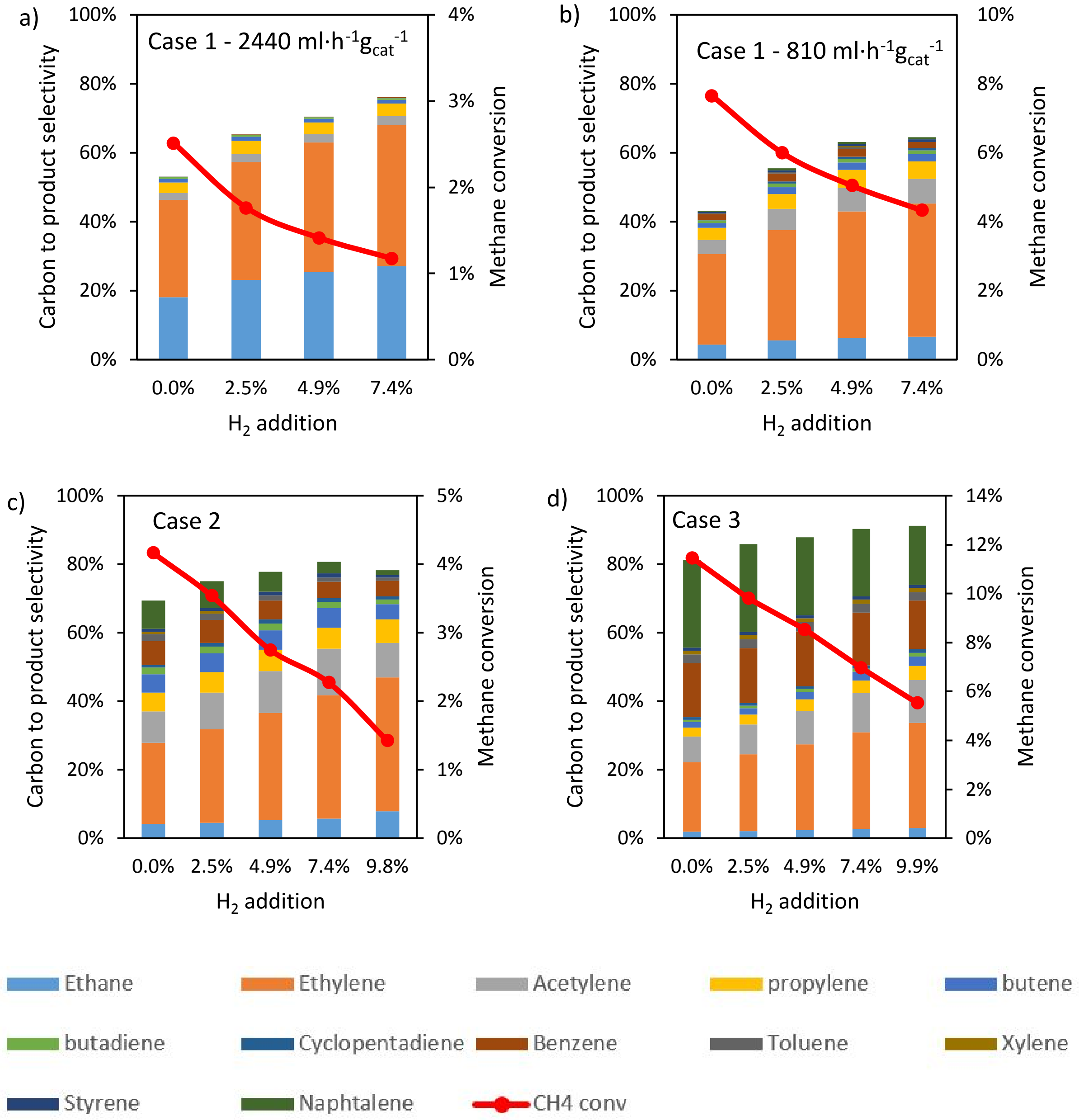


*Figure S1: Carbon to product selectivity and methane conversion when dosing small amounts of hydrogen into the reactant mixture; reactor-zone at 1000 °C, pre-heater at 400 °C; post-heater at 400 °C for (a), (b) and (c), and 1000 °C for (d); (a) and (b) present case 1 from* ***Error! Reference source not found.****, (c) presents case 2 and (c) presents case 3, Space velocity: 2440 ml·h*$^{-1}$*g*$_{cat}$$^{-1}$ *for (a), (c) and d); Space velocity: 810 ml·h*$^{-1}$*g*$_{cat}$$^{-1}$ *for (b)).*

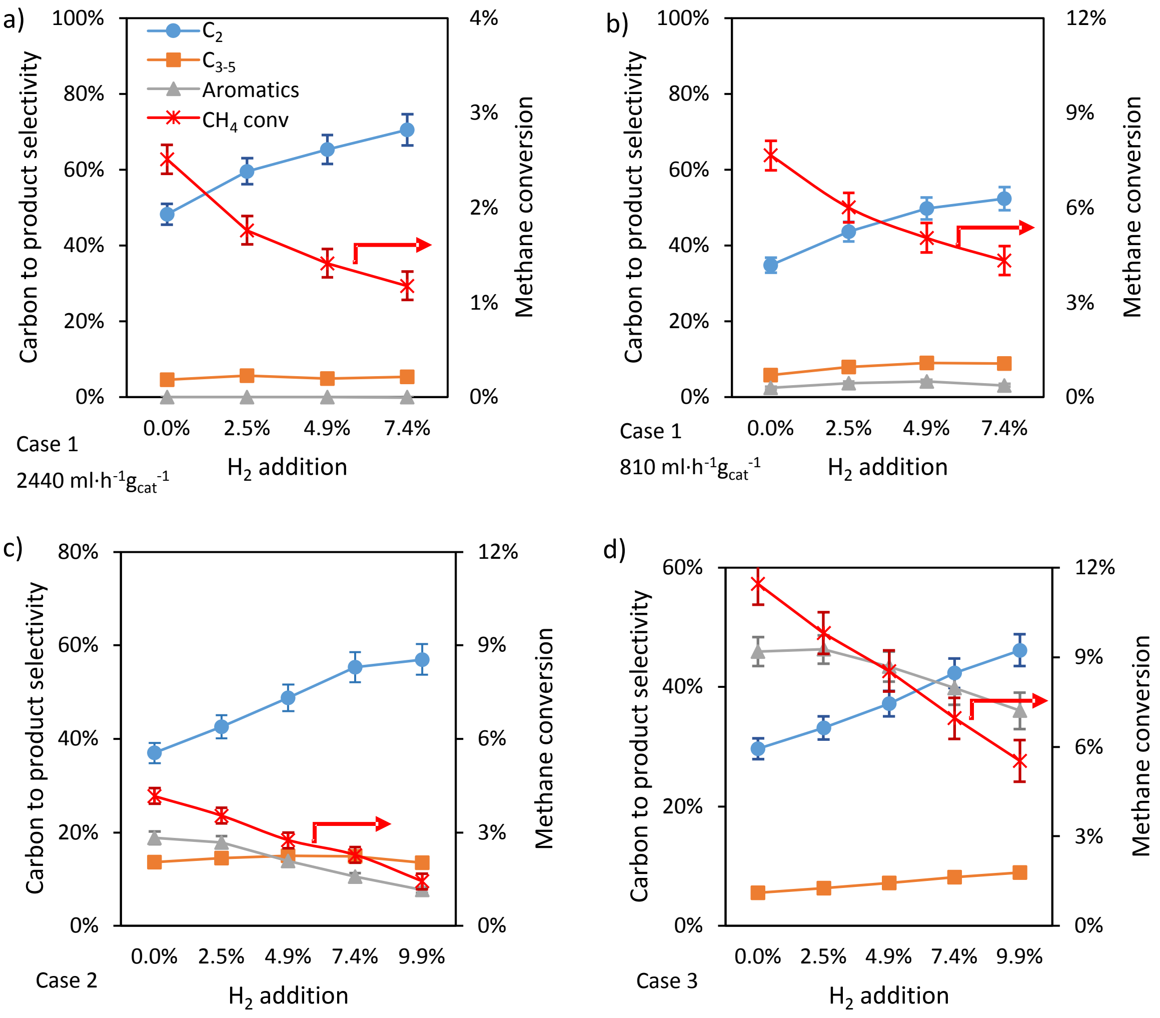


*Figure S2: Carbon to product selectivity over major product groups and methane conversion when dosing small amounts of hydrogen into the reactant mixture; reactor-zone at 1000 °C, pre-heater at 400 °C; post-heater at 400 °C for (a), (b) and (c), and 1000 °C for (d); (a) and (b) present case 1 from* ***Error! Reference source not found.****, (c) presents case 2 and (c) presents case 3, Space velocity: 2440 ml·h$^{-1}$g$_{cat}^{-1}$ for (a), (c) and d); Space velocity: 810 ml·h$^{-1}$g$_{cat}^{-1}$ for (b)).*

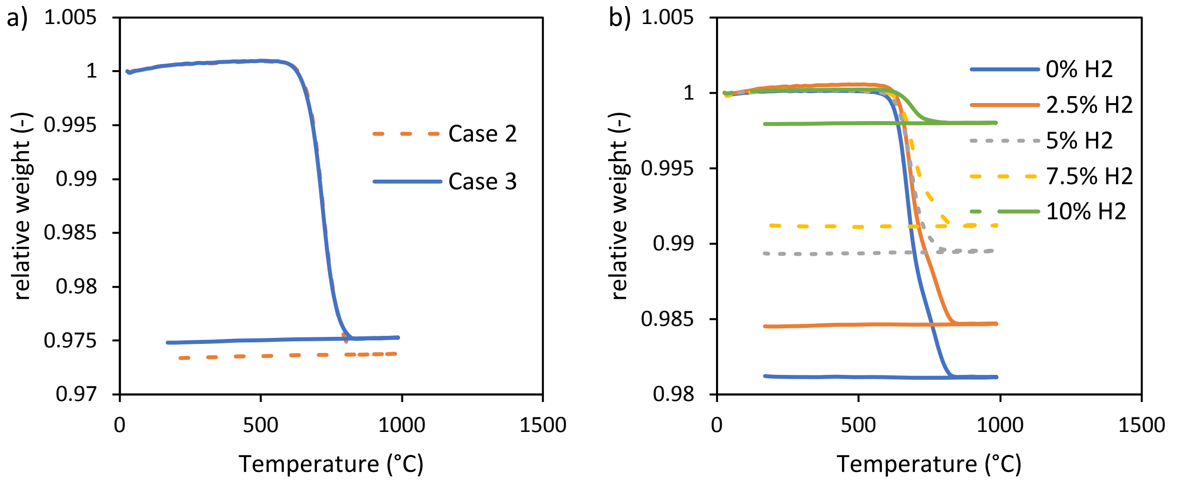


*Figure S3: TGA analysis of the spent catalyst samples, pre-heater at 400°C and reactor-zone at 1000°C, Space velocity: 2440 ml·h$^{-1}$g$_{cat}^{-1}$, (a) Case 2 using post-heater at 400°C and Case 3 using post-heater at 1000°C. (b) experiments using fixed inflow concentrations with post-heater at 400°C.*
